# Investigating the electronic properties of graphene oxide functionalized with benzoic acid

**DOI:** 10.1038/s41598-025-22839-w

**Published:** 2025-10-31

**Authors:** Hanan Elhaes, Medhat A. Ibrahim

**Affiliations:** 1 Physics Department, Faculty of Women for Arts, Science and Education, Ain Shams University, Cairo, 11757 Egypt; 2 Spectroscopy Department, National Research Centre, 33 El-Bohouth St., Dokki, Giza 12622 Egypt; 3 Molecular Modeling and Spectroscopy Laboratory, Centre of Excellence for Advanced Science, National Research Centre, 33 El-Bohouth St., Dokki, Giza 12622 Egypt

**Keywords:** GO, Benzoic acid, DFT: B3LYP/6-31 g(d, p), Electronic properties, Chemistry, Materials science, Nanoscience and technology, Physics

## Abstract

This study employs density functional theory (DFT) to investigate the intricate non-covalent interactions between graphene oxide (GO) and benzoic acid (BA), offering a crucial theoretical foundation for the rational design of advanced GO-based composites. Using the B3LYP/6-31 g(d, p) model, we’ve demonstrated that the functionalization of GO with one or two units of BA leads to a remarkable modification of its electronic properties. Our findings reveal a complex, multifaceted interaction characterized by hydrogen bonding, dative bonding, and π-π stacking, as confirmed by Molecular Electrostatic Potential (MESP) and Quantum Theory of Atoms in Molecules (QTAIM) analyses. This synergistic bonding mechanism alters the electronic structure, leading to a modified HOMO-LUMO gap and enhanced charge transfer. The Density of States (DOS) analysis confirms the creation of new hybrid orbital features and a reduction in electrical conductivity, which is a key property for many electronic applications. Furthermore, the calculated infrared (IR) and Raman spectra corroborate the formation of these new composite structures. These results provide fundamental insights into the tunable electronic properties of GO/BA composites, making them highly promising for applications requiring precise control over charge transport. This work lays the groundwork for the development of next-generation sensors, catalysts, and electronic devices by showing how simple molecular functionalization can unlock new functionalities in graphene-based materials.

## Introduction

Graphene-based systems are considered among the hottest topic of research owing to their unique electronic properties. Such properties, besides their high surface area and their excellent electrical conductivity allow these systems to be tunable^1,2^. Graphene oxide (GO) is one of the important members of graphene-based systems, it has abundant oxygen- rich functional groups such as hydroxyl, epoxy, carbonyl and carboxyl. This in turn enable GO, to be easily functionalized with different nanomaterials forming new composites with specific features^3–5^. On the other hand, benzoic acid BA is a white, crystalline, aromatic carboxylic acid naturally found in some plants and animals, and could be also produced by some microorganisms. It’s widely applied in many fields such as a food preservative, in cosmetics, hygiene products, and pharmaceuticals^6,7^.

Graphene can be functionalized with benzoic acid derivatives such as p-aminobenzoic acid using the diazonium-grafting method, allowing the basal planes to be extended by benzoic acid groups^8,9^. Graphene oxide (GO) interacts with benzoic acid primarily through a combination of non-covalent interactions and, in some cases, can be chemically modified with benzoic acid derivatives. Understanding these interactions is crucial for tailoring GO-based materials for various applications^10,11^.

As benzoic acid possesses a carboxyl group and a phenyl ring, it can form hydrogen bonds with the oxygen functionalities on the GO surface^12^. Therefore,, benzoic acid could be used to covalently functionalize graphene oxide. This involves reactions between the carboxyl groups of the benzoic acid and the carboxyl or hydroxyl groups on the GO surface^13^, this include possible adsorption of benzoic onto the surface of graphene oxide. Studies have shown that the adsorption capacity is influenced by factors like pH and the specific surface chemistry of the GO. It is stated that, the π-stacking ability of the aromatic compound often plays a dominant role in regulating adsorption capacity^14^. Also, the possible interaction with benzoic acid can modify the surface properties of GO. For example, if covalent functionalization occurs, it can introduce new chemical groups, altering hydrophilicity/hydrophobicity, reactivity, and dispersibility^15,16^. Functionalized GO, such as with aminobenzoic acid, has shown improved electrochemical behavior for certain applications, like oxygen reduction reactions and supercapacitors, due to the increased density of oxygenated functional groups^17^. In some cases, the interaction of GO with organic molecules like tannic acid (which also contains phenolic and carboxylic acid groups) can mitigate the toxicity of GO, potentially by altering its surface interactions in biological systems^18^.

Graphene oxide frameworks (GOFs) obtained by self-assembly of benzoic acid functionalized GO with metal ions (like Zn(II)) have been developed for environmental sensing applications, such as the electrochemical oxidation of nitrophenol isomers^19^. It is also reported that, a chemosensor is considered among the several applications for GO/BA composite^20^.

Density Functional Theory (DFT) is a powerful quantum mechanical tool for investigating the electronic structure of molecules and materials^21^. It is a valuable method for gaining atomistic and electronic insights into many properties and interactions^22^. It’s typically used to elucidate reactivity;, adsorption mechanisms,; spectroscopic interpretation,  and to understanding structure-property relationships^23,24^.

DFT could provide researchers with some parameters used to characterize the electronic structure, chemical behavior and reactivity of molecules and materials^25,26^. Such parameters are helpful to predict how the molecules will interact with each other as well as with their surrounding molecules^27–29^.

The motivation for this work is to explore how the electronic properties of graphene oxide (GO) can be precisely tuned by functionalizing it with a common organic molecule, benzoic acid (BA). Understanding this interaction is crucial for developing GO-based materials with tailored functionalities. The novelty lies in the detailed, atomistic-level investigation of the specific bonding mechanisms-including hydrogen bonding, π–π stacking, and dative bonding-between GO and BA. By studying both GO/BA and GO/2BA complexes, this work goes beyond simple adsorption to demonstrate how the stoichiometry of functionalization can be used to control electronic properties like the HOMO-LUMO gap and charge transfer. This provides a fundamental theoretical basis for the rational design of next-generation sensors, catalysts, and electronic devices. So that, the present work is carried out to study the functionalization of GO with benzoic acid BA. Benzoic acid is supposed to interact with GO through either OH or COOH or both of them. Electronic properties for GO, BA, GO/BA and GO/2BA were elucidated with DFT at B3LYP/6-31 g(d, p) level.

## Molecular modeling calculations

### Building model molecules

**Fig. 1:**
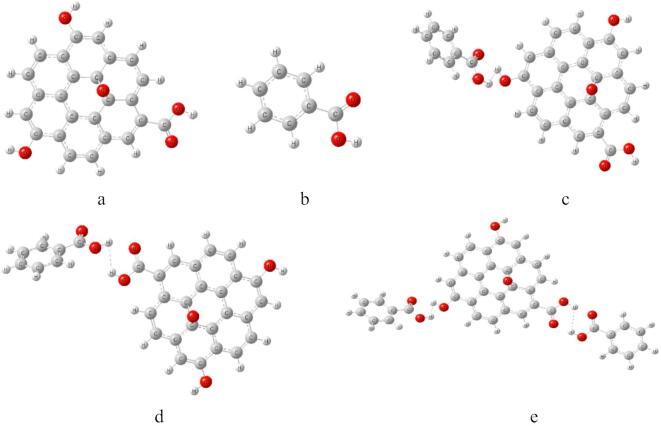
The studied model structures whereas; (**a**) GO, (**b**) BA, (**c**) GO/BA- OH, (**d**) GO/BA- COOH, and (**e**) GO/2BA- OH and COOH.

Graphene oxide GO model is indicated in Fig. 1a, the surface of GO shows the existence of oxygen atom forming epoxide. The terminal of GO was attached with two OH groups and one COOH group. Another model for aromatic hydrocarbon is indicated in Fig. 1b, which is the benzoic acid abbreviated as BA. Possible interaction between GO and BA is indicated in Fig. 1c, whereas the interaction took place via weak interaction between BA and OH of the GO model. Another possible interaction between GO and BA could be through the weak interaction with COOH of the GO as shown in Fig. 1d. Gathering the two possible interactions between GO and BA, the model in Fig. 1e indicated that, two BA units are weakly interacted with GO throughout OH and COOH respectively.

### Details of computations

GO, BA and GO/BA with different configuration indicated in Fig. 1, were subjected to calculation using G09 software^30^ which implemented at the Molecular Modeling and Spectroscopy Laboratory, Centre of Excellence for Advanced Science, National Research Centre, Egypt. The structures were optimized with density functional theory at B3LYP^31–33^ using6–31 g(d, p)basis set. Frequency and Raman were calculated at the same level of theory. A clear justification for the chosen level of theory is crucial for the reproducibility and credibility of DFT results. The B3LYP/6–31 g(d, p) model is a standard and popular choice for organic systems due to its balance of computational efficiency and accuracy for a wide range of properties. Some important parameters were also calculated as the total dipole moment, HOMO/LUMO band gap energy, molecular electrostatic potential MESP and density of states DOS. Energy descriptors were calculated based on HOMO and LUMO values such as ionization potential (I), electronic affinity (A), chemical potential (µ), chemical hardness (η), absolute chemical softness (S) and electrophilicity index (ω)^34,35^, the calculated descriptors were derived as indicated in the following equations:

$${\text{I }} = - {\text{E}}_{{{\text{HOMO}}}}$$

$${\text{A = E}}_{{{\text{LUMO}}}}$$

$$\mu = - {\text{(I + A)/2}}$$

$$\eta = ({\text{I}} - {\text{A}})/2$$

$${\text{S = 1/}}\eta$$

$$\omega = \mu ^{2} /2\eta$$

To check the stability of the studied structures the quantum theory of atoms in molecules QTAIM calculations were conducted with both Multiwfn and VMD software^36,37^.

## Results and discussions

### TDM and HOMO/LUMO energy gap

Table 1 presented the total dipole moment (TDM) in Debye and HOMO/LUMO energy gap (ΔE) in eV for the studied systems. The TDM was 4.119144 Debye for GO, and was 1.915365 Debye for BA.  When GO interacted with BA through OH group,  the TDM was 4.207083 Debye, which then increased to 4.893122 Debye for interaction through the COOH group.. As GO interacted with two BA units the TDM decreased to be 2.686371 Debye. Regarding the HOMO/LUMO energy gap (ΔE), it was 2.939397032 eV for GO then was 5.780015956 eV for BA. The interaction between GO and BA through OH, COOH and through both of them keep the ΔE almost unchanged.

**Table 1:** Calculated total dipole moment (TDM) in Debye and HOMO/LUMO energy gap (ΔE) in eV for the studied systems.

Structures	TDM (Debye)	ΔE (eV)
GO	4.119144	2.939397032
BA	1.915365	5.780015956
GO/BA - OH	4.207083	2.945927816
GO/BA- COOH	4.893122	2.910008504
GO/2BA - OH and COOH	2.686371	2.91025923

### Frontier molecular orbitals and density of states

**Fig. 2:**
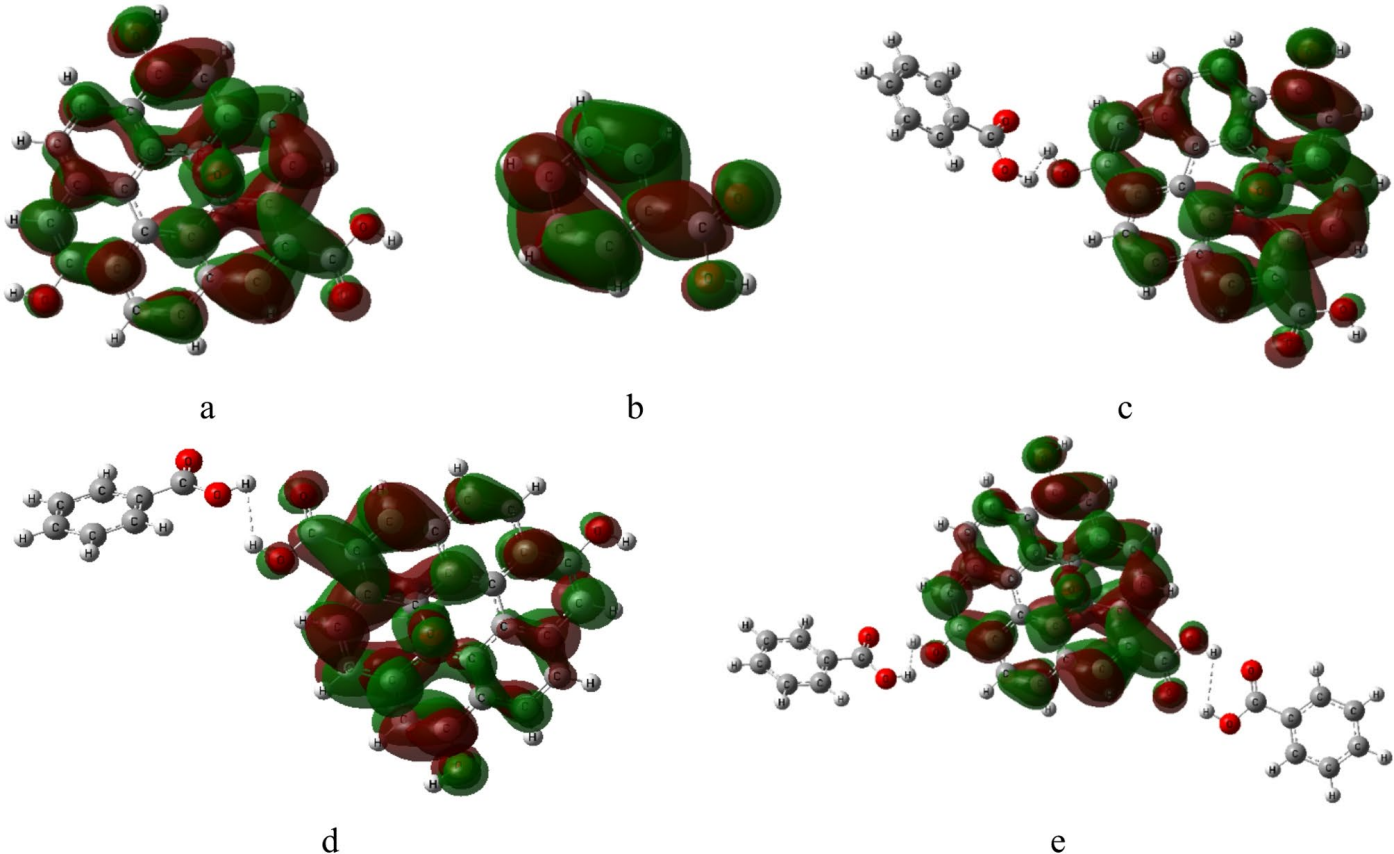
Frontier molecular orbitals calculated at B3LYP/6–31 g(d, p)level for the studied structures whereas; (**a**) GO, (**b**) BA, (**c**) GO/BA- OH, (**d**) GO/BA- COOH, and (**e**) GO/2BA- OH and COOH.

Figure 2 presented the Frontier Molecular orbitals calculated at B3LYP/6–31 g(d, p) level for the studied structures whereas the green color for HOMO red colors for LUMO. The HOMO/LUMO for GO. Figure 2a,b shows the HOMOLUMO orbitals for both GO and BA for GO interacted with BA through OH as shown in Fig. 2c and through COOH as shown in Fig. 2d, the HOMO/LUMO are distributed along the GO. The same is regarded as GO interacted with two BA units as shown in Fig. 2e, the HOMO/LUMO is distributed through the GO.

It is clear that, the interaction between GO and BA modifies their electronic structures, leading to a reduced HOMO-LUMO gap and enhanced charge transfer. These changes are crucial for applications in sensors, catalysis, and electronic devices, where tunable electronic properties are desired^8^.

**Fig. 3:**
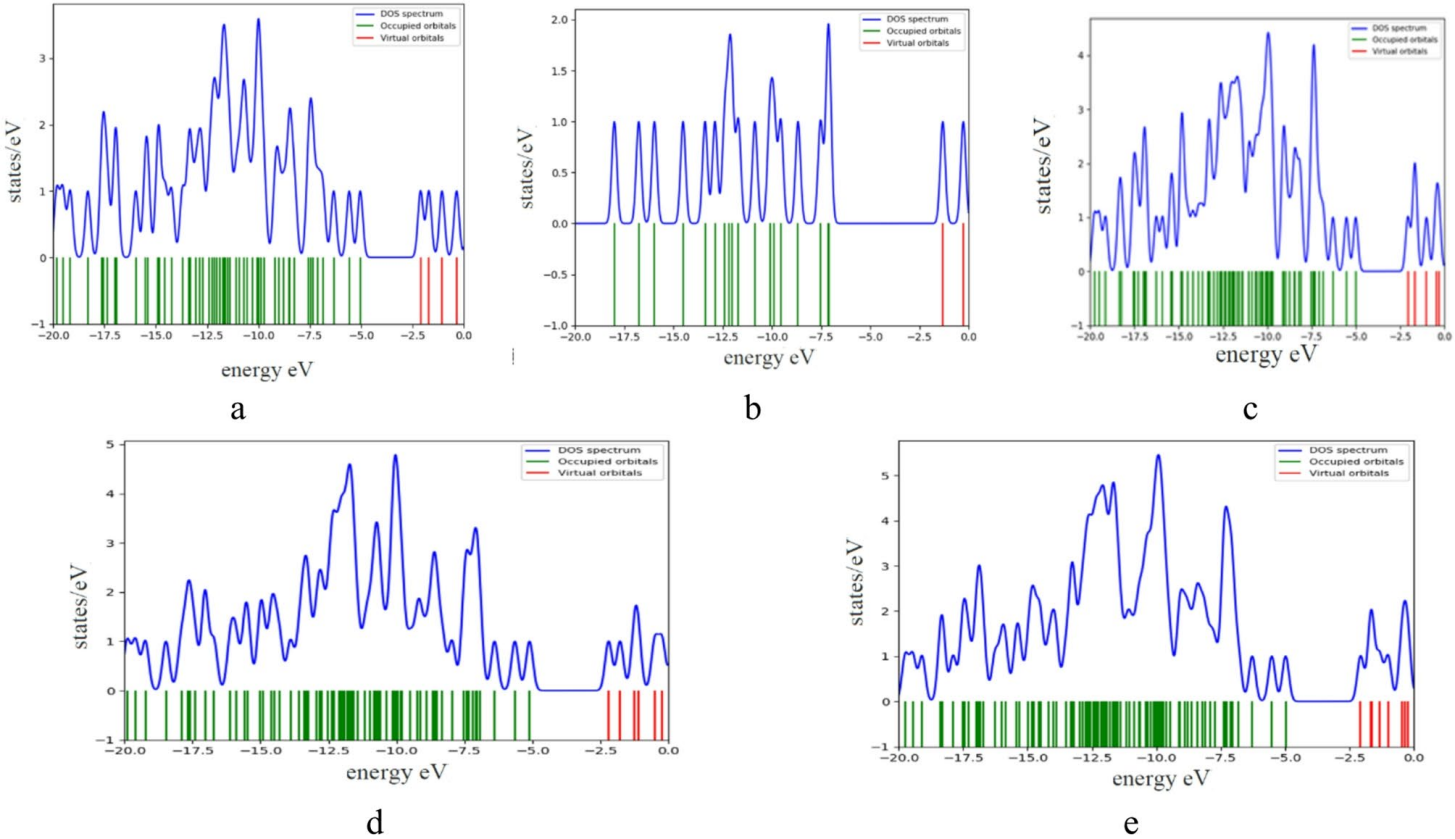
Calculated density of states DOS for the studied structures whereas; (**a**) GO, (**b**) BA, (**c**) GO/BA- OH, (**d**) GO/BA- COOH, and (**e**) GO/2BA- OH and COOH.

More insight into the HOMO/LUMO orbitals could be described with the help of density of states DOS which is plotted for the studied structure as indicated in Fig. 3a–e.

As shown in Fig. 3a, the DOS for GO, whereas the blue line is the total DOS spectrum, the green represented the energies of occupied molecular orbitals. While the red is the energies of unoccupied (virtual) molecular orbitals. The blue DOS line shows multiple sharp and broad peaks in this region, indicating a high density of states from many overlapping orbitals. This region represents the valence band. There is a clear gap, impling that graphene oxide has a finite band gap. The gap suggests semiconducting behavior, which arises due to the oxidation of graphene.

Sharp peaks may indicate localized electronic states; possibly from functional groups (OH and COOH) the presence of a gap implies reduced electrical conductivity, which is consistent with known properties of graphene oxide.

Figure 3b, showed the DOS for benzoic acid, the DOS spectrum (blue curve) shows a series of sharp and distinct peaks, a noticeable energy gap exists, which is typical for organic molecules. The electronic structure of BA reflects contributions from aromatic π-system in the benzene ring; carboxylic group, which has both lone-pair orbitals and π-bonding. On the other hand, the HOMO is likely a π orbital delocalized over the aromatic ring, while the LUMO is likely a π* orbital or may involve antibonding character from the carboxyl group.

Figure 3c, presented the DOS for GO interacted with BA through OH. When benzoic acid (BA) interacts with graphene oxide (GO), the combined DOS reflects contributions from both subsystems-featuring GO’s broad valence-band characteristics and BA’s discrete molecular states-plus interaction-induced features. While I couldn’t find a direct DOS plot of GO/BA, studies on aromatic acid adsorption on GO help interpret your combined DOS. The GO provides a broad valence band structure rich in O-2p features, while BA contributes sharp molecular peaks. BA/GO creates new coupling-induced features, particularly in the mid-binding energy range, the energy gap remains, indicating preserved semiconducting/insulating behavior^38^. Figure 3d, indicating almost the same behavior regarded in Fig. 3c.

Figure 3e, presented the DOS for GO interacted with two BA units through OH and COOH. It is clear that, adding more benzoic acid amplifies the discrete molecular signature atop GO’s broad DOS envelope. Non-covalent π–π stacking and hydrogen bonding strengthen electronic coupling, creating new hybridization features. Crucially, the energy gap remains intact-electric conductivity doesn’t turn metallic with adsorption.

Correlating both HOMO/LUMO with the DOS one can notice that the π–π stacking and hydrogen bonding, creates new hybridization features and amplifies the molecular signature of BA. This analysis links the electronic states directly to the components and the type of interaction between them, providing a more detailed explanation of the observed DOS plots.

### Molecular electrostatic potential MESP

**Fig. 4:**
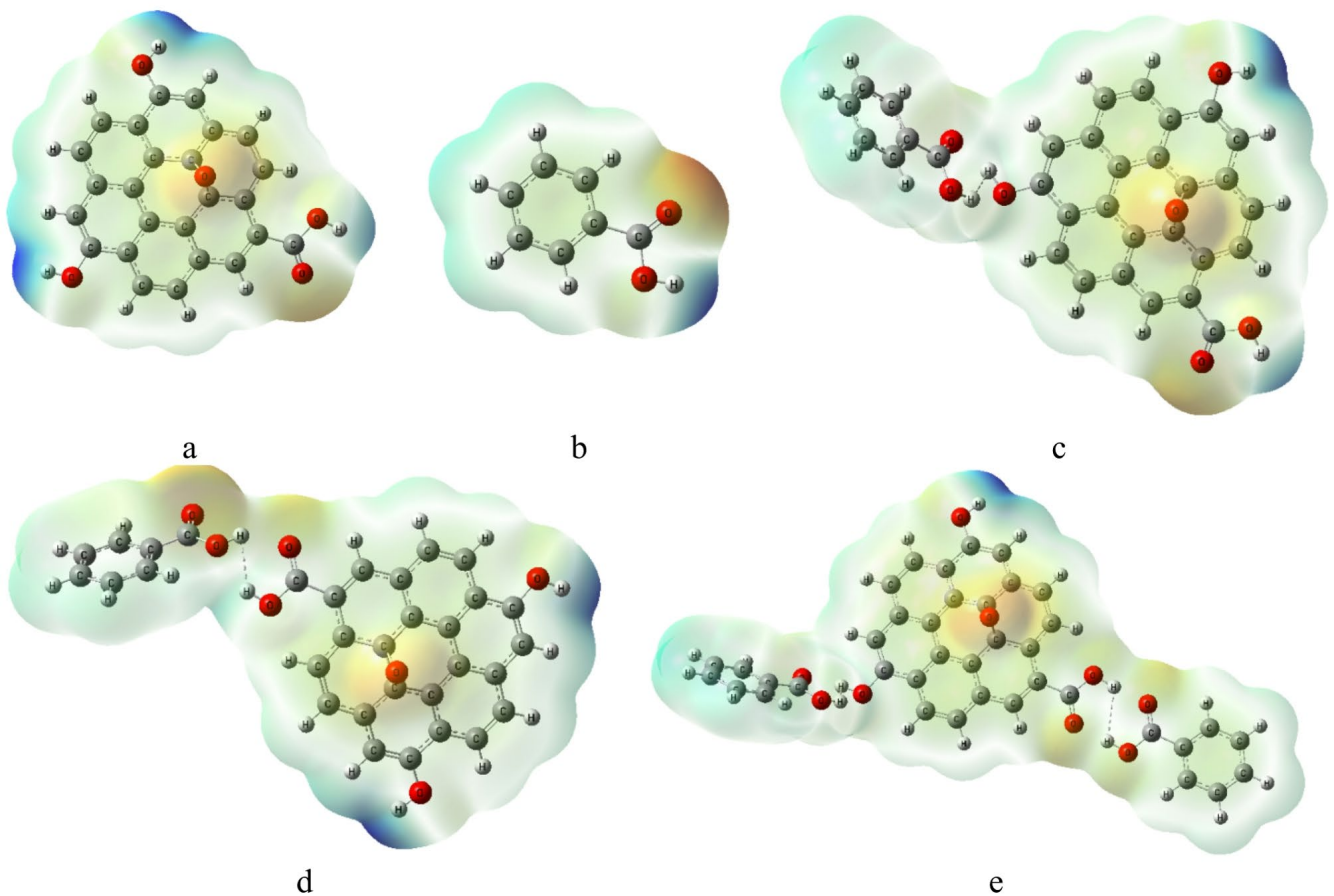
MESP calculated at B3LYP/6–31 g(d, p) level for the studied systems whereas; (**a**) GO, (**b**) BA, (**c**) GO/BA- OH, (**d**) GO/BA- COOH, and (**e**) GO/2BA- OH and COOH.

Molecular Electrostatic Potential MESP show the color scheme as red /orange (high electron density, negative potential): which is likely to donate electrons, suited for interacting with electrophiles or forming hydrogen bonds. While blue /green (lower electron density, positive potential).Which indicated that, it is more electron-deficient and potentially electrophilic, accepting electron density from nucleophiles. The MESP for GO indicated in Fig. 4a, the Figure showed that, below and around the GO sheet, the neutral green/white region reflects delocalized π-electron character, typical of sp^2^-hybridized carbons in graphene oxide. As a result of introducing oxygen-containing groups disrupts this delocalization marginally, creating local polarization. MESP for BA indicated in Fig. 4b, show reactive area close to the COOH group. GO interacted with BA as shown in Fig. 4c, the surface at and around oxygen containing groups are reactive. The same is regarded for GO interacted with BA throughout COOH as shown in Fig. 4d^39^.

Finally, the MESP for GO interacted with two BA is indicated in Fig. 4e. The highly negative O centers (deep red areas) align with hydrogen donors from BA’s –COOH groups and GO’s –OH, indicating strong hydrogen bonds as the primary mode of adsorption. The neutral to slightly positive areas over GO’s π-plane and BA’s rings suggest ideal orientation for π–π stacking, reinforcing non-covalent adhesion^40^.

### Theory of atoms in molecules QTAIM

**Fig. 5:**
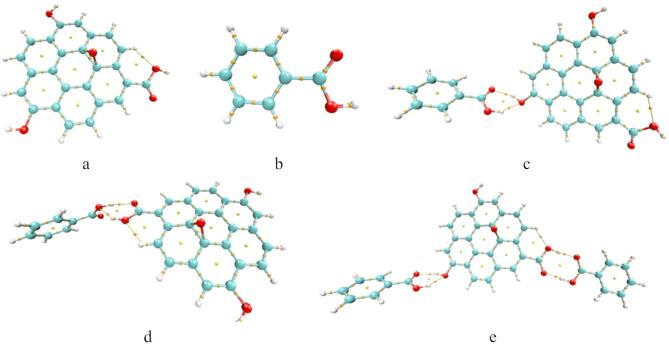
Calculated QTAIM topology for the studied structures whereas; (**a**) GO, (**b**) BA, (**c**) GO/BA- OH, (**d**) GO/BA- COOH, and (**e**) GO/2BA- OH and COOH.

Figure 5a presented the Quantum Theory of Atoms in Molecules (QTAIM) topology for GO. The map shows that, the electron density has saddle points between atoms. The properties at these points help determine bond type and strength, whether interactions are dative, covalent, ionic, or dispersive^41^. QTAIM reveals multiple non-covalent bonding sites; each type shows distinct electron-density signatures. This topology underpins the strong yet non-destructive adsorption, which matches what’s observed in similar systems including GO–tannic acid and GO–amino acid complexes^14^. As shown in Fig. 5b, the QTAIM map for BA confirms a multipoint, synergistic interaction regime.  When GO interacts with BA as indicated in Fig. 5c, the QTAIM topology analysis reveals that the interaction between GO and benzoic acid molecules is multifaceted, involving π–π stacking, hydrogen bonding, and dative bonding. These interactions collectively contribute to the stability and functionality of the GO–benzoic acid complex. The same could be also noted for the other GO/BA interactions indicated in Fig. 5d.

Figure 5 presented the calculated QTAIM topology for GO/2BA interacted throughout both OH and COOH. The QTAIM topology for GO interacting with two BA units reveals that the interaction between GO and benzoic acid molecules is multifaceted, involving π–π stacking, hydrogen bonding, and dative bonding. These interactions collectively contribute to the stability and functionality of the GO–benzoic acid complex, which is crucial for various applications such as catalysis, sensing, and environmental remediation^42^.

### Spectroscopic analyses

**Fig. 6:**
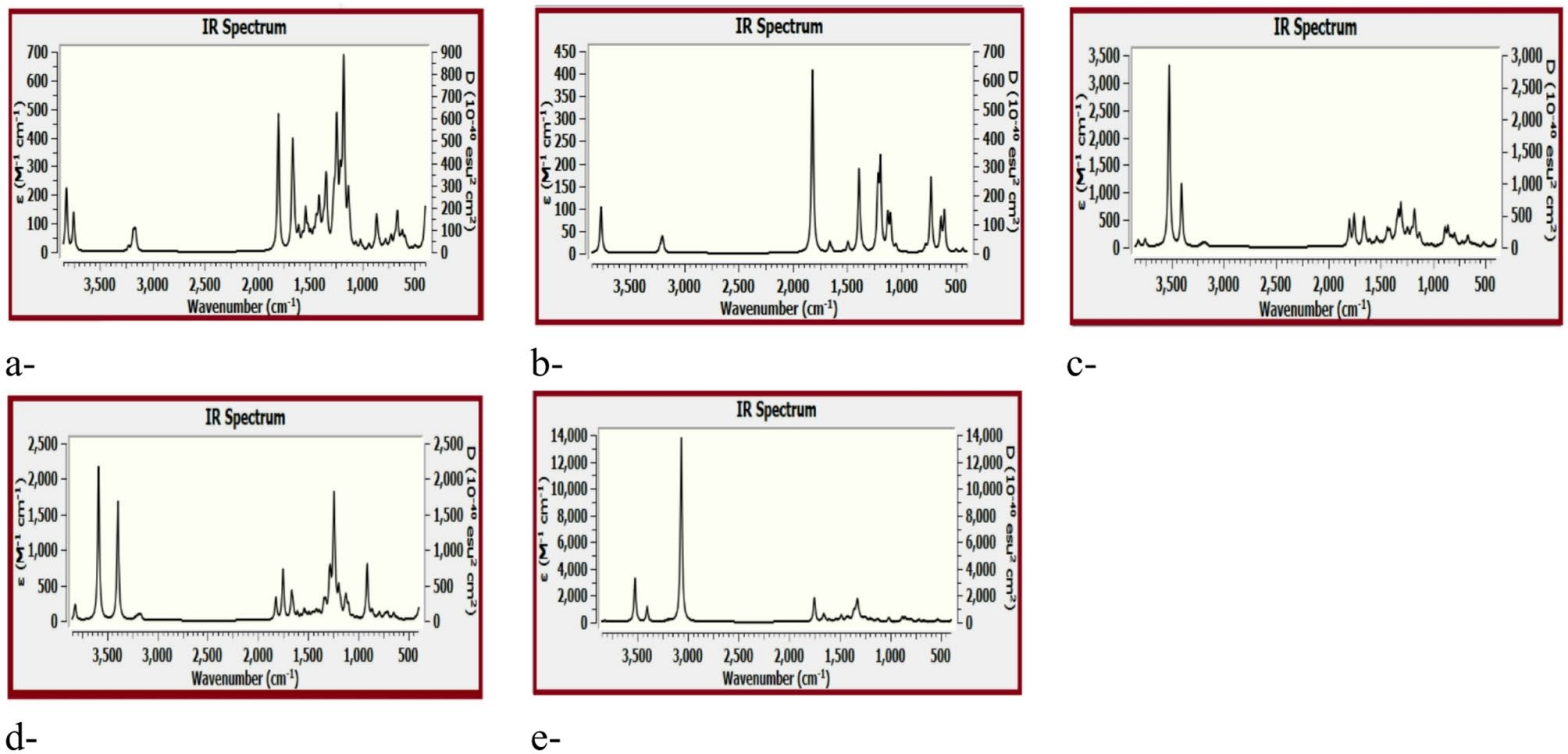
The IR spectra for the studied model structures whereas; (**a**) GO, (**b**) BA, (**c**) GO/BA- OH, (**d**) GO/BA- COOH, and (**e**) GO/2BA- OH and COOH.

Spectroscopic analyses are discussed in terms of both IR and Raman for the studied structures. It is worth to mention that assignment of the studied bands are aided by the GaussView 5.0^43^. software through animating each band and/or shift for either IR or Raman. Figure 6 presented the IR spectra for the studied model structures whereas; (a) GO, (b) BA, (c) GO/BA - OH, (d) GO/BA COOH, and (e) GO/2BA- OH and COOH. The IR spectrum of GO indicated in Fig. 6a shows several absorption bands, the O–H stretching band is located at 3752.01 to 3752.01 cm^− 1^ (3823.51 corresponding to COOH; 3819.14 and 3752.01 corresponding to OH). C–H stretching band is located at 3177.28 cm^− 1^. The C=O stretch band is located at 1800.64 cm^− 1^, while the band at 1667.88 ~ 1651.78 cm^− 1^ is assigned for C=C stretching, C–O stretching is located at 1370.67 ~ 1342.01 cm^− 1^, the C–H out-of-plane bending is calculated at 979.01 ~ 776.39 cm^− 1^.

The IR spectrum of BA is indicated in Fig. 6b; the spectrum shows the OH stretching at 3766.64 cm^− 1^. The bands at 3206.14 ~ 3196.27 cm^− 1^ are assigned as aromatic C–H band. While the band at 1819.73 cm^− 1^ is assigned as C=O stretching. The C=C stretching vibrations is located at 1492.86 cm^− 1^, C–O band coupled with OH is located at 1194.97 cm^− 1^.

Figure 6c showed the IR of GO/BA interacted through the OH group of GO. The OH bands arise from both OH of GO and OH of COOH is located at 3818.84 ~ 3404.48 cm^− 1^. The band around 3190.24 ~ 3160.37 cm^− 1^ is assigned for the aromatic C–H stretching vibrations from the benzene ring of benzoic acid. The bands 1800.04, 1754.60 cm^− 1^ is assigned for C=O stretching vibrations from GO and BA respectively. The C=C stretching from the aromatic rings of benzoic acid is located at 1661.27 cm^− 1^, the same band for GO is located at 1651.30 cm^− 1^. The O–H bending of carboxylic acid groups (in-plane bending) coupled with C–H bending vibrations is located at 1604.95, 1539.38 cm^− 1^. The band at 1437.83, 1412.69 cm^− 1^ is the C–O stretching vibrations from BA and GO. The C–O–C stretching presents in graphene oxide exhibit a band at 1016.74 cm^− 1^. Finally, another OH bands are located at 884.96 ~ 856.63 cm^− 1^, this band is for BA and GO as well.

The IR of GO/BA interacted through the COOH is indicated in Fig. 6d. the IR spectrum is almost gathering both IR spectra for BA and GO. It is nearly similar to that in Fig. 6c with complex patterns in the fingerprint region. The OH band in Fig. 6d is split into two bands. This may be due to the bonded OH in case of interaction through OH as shown in Fig. 6c the C=O stretching is stronger Fig. 6d as compared with Fig. 6c. As far as GO interacted with two BA through both OH and COOH the IR spectrum is indicated in Fig. 6e.

The spectrum shows several peaks at different wavenumbers, indicating the presence of various functional groups gathering both of GO and BA.

**Fig. 7:**
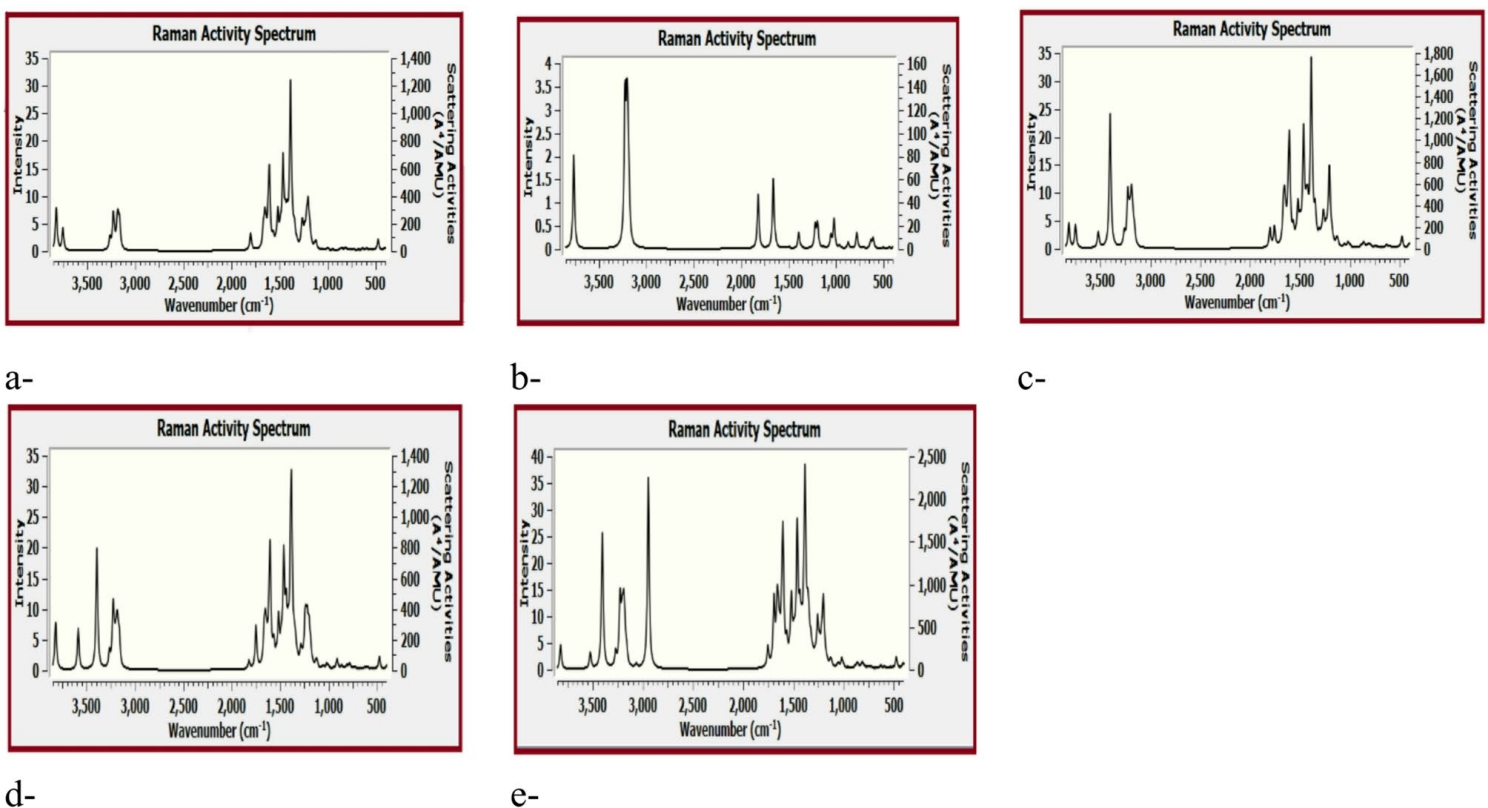
The Raman spectra for the studied model structures whereas; (**a**) GO, (**b**) BA, (**c**) GO/BA- OH, (**d**) GO/BA- COOH, and (**e**) GO/2BA- OH and COOH.

Figure 7 presented the Raman spectra for the studied model structures whereas; (a) GO, (b) BA, (c) GO/BA- OH, (d) GO/BA- COOH, and (e) GO/2BA- OH and COOH. The overall interpretation is that, the Raman shift is gathering those of GO and BA.

The key features observed in this spectrum Strong and sharp peaks are characteristic of carbon–carbon stretching vibrations, especially in aromatic and sp2 hybridized carbon systems.

A strong intense peak around 1580–1600 cm^− 1^ is assigned as G-band is indicative of the graphitic mode (E2g ​phonon mode) of sp2 carbon atoms, which feature of graphene and related materials.

A peak around 1350 cm^− 1^ which the D-band, though not as prominent as the G-band in this specific spectrum, but present as a shoulder or distinct peak, points to defects, disorder, or sp3 hybridized carbon within the graphene structure.

Other peaks can arise from the skeletal vibrations of the benzoic acid units, including C=C stretching of the aromatic ring and C=O stretching if it’s Raman active.

In the 2800–3100 cm^− 1^ region, located sharp peaks which are characteristic of C–H stretching vibrations. The presence of several distinct peaks suggests different types of C–H bonds, likely from both aromatic BA and possibly some aliphatic C–H. The strongest peak in this region is around 3000 cm^− 1^.

Bands around 3400–3500 cm^− 1^ are similar to the previous Raman spectrum; peaks in this region can be attributed to O–H stretching vibrations. These are often weaker in Raman compared to IR, their presence indicates hydroxyl groups.

Bands below 1000 cm^− 1^ are typically correspond to various bending vibrations such as C–C–C bending, C–H out-of-plane bending, and skeletal modes of the molecules.

Comparing to the Raman spectra of GO/BA, strong G-band, and C–H stretching region also appears more resolved with multiple distinct peaks corresponding to GO interacted with two BA units. This is due to the different structural arrangement between graphene oxide and benzoic acid.

## Conclusion

DFT: B3LYP/6–31 g(d, p) was used to model GO; BA; GO/BA and GO/2BA. In terms of both TDM and HOMO/LUMO energy the studied composites are reactive which can be explained in terms some electronic parameters such as in the following.

The DOS for GO/BA shows localized electronic states; which may reduce electrical conductivity. The DOS for GO/2BA indicated new hybridization features. While the energy gap remains intact-electric conductivity doesn’t turn metallic with adsorption.

The MESP for GO/2BA shows highly negative O centers align with hydrogen donors from functional groups in both BA and GO, indicating strong hydrogen bonds. The neutral to slightly positive areas over GO’s π-plane and BA’s rings suggest ideal orientation for π–π stacking.

The QTAIM reveals that, GO/BA composite is multifaceted, involving π–π stacking, hydrogen bonding, and dative bonding.

Vibrational analyses revealed composite formation as each feature of GO and BA is gathered forming those for GO/BA.

It could be concluded that, the interaction between GO and BA is a complex interplay of non-covalent forces, and in some cases, covalent bonding. These interactions, supported by vibrational analysis that showed the distinct features of both GO and BA in the composite, enable the tuning of GO’s electronic properties. This unique tunability makes the GO/BA and GO/2BA composites promising candidates for various applications, including sensing, environmental remediation, and the development of advanced materials.

## Data Availability

The data that support the findings of this study are available from the corresponding author upon reasonable request.
